# Biological variation of baseline serum cortisol concentration in healthy dogs, part 2: clinical applications

**DOI:** 10.3389/fvets.2026.1805888

**Published:** 2026-05-15

**Authors:** Sam Wicker, Sydney Craig, Julia D. Albright, Cary Springer, Kathleen Freeman, Luca Giori

**Affiliations:** 1 College of Veterinary Medicine, Biomedical and Diagnostic Sciences, University of Tennessee, Knoxville, TN, United States; 2 College of Veterinary Medicine, Small Animal Clinical Sciences, University of Tennessee, Knoxville, TN, United States; 3 Office of Innovative Technologies, Research Computing Support, University of Tennessee, Knoxville, TN, United States; 4 Veterinary Information Network, Davis, CA, United States

**Keywords:** baseline cortisol, biological variation, canine (dog), endocrinology, healthy dogs

## Abstract

**Introduction:**

Biological variation tools can be used to quantify uncertainty inherent in laboratory measurements and assist veterinarians in clinical decision making.

**Methods:**

Biological variation tools for interpretation of baseline cortisol concentrations in dogs are calculated using data obtained during part 1 of this study.

**Results:**

Expanded measurement uncertainty and bidirectional dispersion with 95% probability for baseline cortisol are 13.7 and 64.9% respectively, and these values can be used to establish a “grey-zone” around a population-based reference interval limit to guide clinical investigations. Index of individuality is intermediate (1.14), and forty-two samples would be needed to estimate an individual dog’s homeostatic setpoint for cortisol. These values support current recommendations to interpret baseline cortisol concentrations in a broader clinical context utilizing ancillary testing when appropriate. The unidirectional reference change value for baseline cortisol is 77.3% with 95% probability, which can be helpful in determining the significance of changes in serial measurements.

**Discussion:**

Biological variation tools help define uncertainty around measurement of baseline cortisol concentrations in dogs, encourage an individualized approach to spectrum-of-care, and reinforce current consensus statements that rely on clinical signs combined with dynamic testing for the diagnosis of hyper- or hypoadrenocorticism rather than baseline cortisol concentrations alone.

## Introduction

Baseline cortisol concentrations (prior to dynamic testing) have been used as a screening tool for hypoadrenocorticism in dogs (1–4) and many diagnostic laboratories provide population-based reference intervals (pRI) for this measurement to aid interpretation. While baseline cortisol attempts to identify pathophysiological changes to the homeostatic setpoint (HSP), these measurements are also affected by individual variability around that setpoint, normal variation in HSP between individuals, and analytical variability. Biological variation in baseline cortisol concentrations for healthy, client-owned dogs acclimated to the collection environment and phlebotomist was quantified in part one of this study as the within-individual coefficient of variation (CV_I_) and between-individual (group) coefficient of variation (CV_G_), with the intra-assay analytical coefficient of variation (CV_A_) of the analyzer also reported.

When comparing a measurement to a pRI or the results of prior testing, quantifying uncertainty due to biological and analytical variation helps avoid misinterpretation and inappropriate clinical decisions. This topic has grown increasingly relevant with the expanded use of point-of-care analyzers in general veterinary practices, enabling rapid cortisol measurements but raising concerns over reliance on single numerical values without adequate appreciation of measurement variability.

Several mathematical tools have been described to apply biological variation data to laboratory medicine and data interpretation (5–10). Understanding the expanded measurement uncertainty (EMU) and dispersion of a measurand define “gray-zones” around a measured value that accounts for uncertainty due to analytical and individual variation. Calculating the index of individuality (IoI) and estimating the number of samples required to determine the HSP in an individual can define whether individualized or population-based reference intervals are more appropriate for clinical decision-making. Reference change values (RCV) applied to serial data help differentiate true changes in HSP from changes that may be due to biological variation. Each of these tools provides insights into patient data interpretation in different clinical scenarios. Rather than contributing additional uncertainty surrounding an individual measured concentration, they characterize the uncertainty already present. A veterinarian may utilize these tools when making clinical decisions to contribute to individualized spectrum-of-care.

Our goal in this manuscript is to apply the biological variation of baseline cortisol quantified in part one of this study with these interpretation tools and illustrate their clinical utility. We calculate the expanded measurement uncertainty (EMU) and dispersion of baseline cortisol in dogs and demonstrate how these measures can be used to define a “grey zone” around a pRI limit. We provide an index of individuality and the number of samples needed to estimate the HSP for baseline cortisol and discuss the implications of these values on the establishment and utilization of individualized and population-based reference intervals (pRIs). We illustrate how to calculate the reference change value (RCV) of serial cortisol concentrations and provide examples of how RCV can be used to improve clinical decision making. These tools can be used by veterinarians to aid interpretation of baseline cortisol in dogs, and the findings support current consensus statements encouraging interpretation of cortisol concentrations within the wider clinical picture (11).

## Material and methods

Biological variation data (CV_I_ and CV_G_) for baseline cortisol concentrations in healthy, client-owned dogs from Part 1 of this study were used for the calculations in this manuscript (12). The CV_A_ obtained from the study is also used. As the CV_A_ may vary across laboratories and instruments, veterinarians are encouraged to use the CV_A_ specific to their analyzer wherever possible. The study design, sampling procedures, and hormone analysis methods used are described in detail in Part 1 of this study. Briefly, eighteen healthy client- and student-owned dogs from the University of Tennessee College of Veterinary Medicine were enrolled. Dogs were sampled weekly for six weeks during the summer of 2024, with blood collected at consistent times and processed under standardized conditions. Serum cortisol concentrations were measured in duplicate using a chemiluminescent immunoassay system (Immulite 2000 XPi, Siemens Healthcare Diagnostics) as per the manufacturer’s protocol and with laboratory personnel blinded to dog identity and time points. All procedures were approved by the Institutional Animal Care and Use Committee (protocol # 3042), and informed consent was obtained from all owners. The calculations of expanded measurement uncertainty, dispersion, number of measurements required to estimate the homeostatic setpoint, index of individuality, and reference change values are described below, and summary results are reported in Table 1. Confidence intervals for RCV and IoI were obtained using subject-level bootstrap resampling (5,000 iterations) implemented in R (version 4.5.1). For each bootstrap sample, variance components were re-estimated using mixed-effects models (lme4), and RCV and IoI were recalculated.

**Table 1:** Biological variation tools calculated using biological variation data for baseline cortisol obtained from 18 healthy dogs over a 6 week period.

BV tool	Value
Index of individuality	1.138 (CI: 0.61–1.83)
Number of samples needed to estimate HSP	42
Unidirectional reference change value with 95% probability	77.27% (CI: 55.41–97.77%)
Expanded measurement uncertainty	13.7%
Dispersion	64.93%

HSP (homeostatic setpoint). Calculations for the number of samples needed to estimate HSP utilized a bidirectional z-score for 95% probability and 10% allowable deviation. Calculations for reference change value and dispersion used bidirectional z-scores for 95% probability. Expanded measurement uncertainty was calculated using a coverage factor of 2 for 95% probability. Confidence intervals (95%) are reported for index of individuality and reference change value.

## Biological variation tools

### Expanded measurement uncertainty and dispersion

Many veterinarians are conceptually aware of expanded measurement uncertainty and dispersion even if this terminology is not familiar to them. If a single patient sample is repeatedly tested for the same measurand, most veterinarians would expect the serial results to be close in value but not the exact same due to inconsistencies within the analytical method. This expected variability can be quantified and utilized when practicing evidence-based medicine. Repeatability studies describe analytical variability in terms of standard deviations from the mean value or the coefficient of variation (CV_A_). Variance can be combined with a desired probability to mathematically express a range of uncertainty surrounding a single measurement that accounts for analytical variation. Future measurements are expected to fall within this range at a rate consistent with the designated probability (e.g., 95% of future results). This range of values surrounding a single measurement is called the expanded measurement uncertainty (EMU).

EMU was calculated using a coverage factor (K) of 2 for 95% probability with the following equation (10):


$$\mathrm{EMU}=2*({\mathrm{CV}}_{A}^{2}/100)$$

The CV_A_ for our analyzer in part 1 of this study is 6.86% (95% CI: 6.03–7.92%). Thus, the EMU around a single basal cortisol measurement for our analyzer is 0.137, or 13.7%. In other words, if a veterinarian were to repeatedly measure the cortisol concentration in a single sample, the subsequent results are expected to be within 13.7% of the initial result, 95% of the time. As repeated measurements are performed, the EMU can be continuously revised as the “true” value for the analyte is estimated with progressively greater certainty.

While dispersion is similar to EMU, it represents the uncertainty around a measurement accounting for both analytical variability (CV_A_) and normal biological fluctuations around a homeostatic setpoint in an individual (individual variability, CV_I_). In other words, dispersion describes how much variation is expected in repeated sampling over time due to both normal physiologic fluctuations and analytical variability. Dispersion is particularly useful when interpreting results for measurands that are known to have relatively high CV_I_ (> 6.77%) such as baseline cortisol. Dispersion for a single sample analyzed once was calculated using the following equation (5, 9):


$$\text{Dispersion}=Z*\surd ({\mathrm{CV}}_{A}^{2}+{\mathrm{CV}}_{I}^{2})$$

The Z-score of 1.96 was used to determine bidirectional dispersion with a 95% probability. The CV_I_ for baseline cortisol in healthy dogs in part one was found to be 32.4% (95% CI: 28.2–38.1%), with the dispersion calculated to be 64.9%. If a veterinarian were to repeatedly measure baseline cortisol across several weeks with our analyzer, each additional result is expected to be within 64.9% of the initial measurement, 95% of the time. As with EMU, repeated sampling over time will decrease the dispersion as the estimate of the “true” homeostatic setpoint for the individual is identified with increased confidence. While the calculated dispersion around sequential measures can be reduced with repeated sampling, these calculations are not reported here since interpretation of a single baseline cortisol measurement is the most common scenario encountered in a veterinary practice.

A common clinical application of EMU and dispersion is to provide a “grey-zone” around a population reference interval (pRI) limit or clinical decision cut-point. Many practicing veterinarians have encountered scenarios where a measurand of interest is slightly outside or slightly within the pRI. While additional clinical information or other clinicopathological measures often provide clarity, the clinical significance of this finding can occasionally become confusing. EMU and dispersion can be used to establish an objective, evidence-based “grey-zone” around the pRI limits to account for variability due to analytical and biological variation. Concentrations that fall within these limits are of uncertain significance when accounting for biological and analytical variability. Concentrations outside of the EMU or dispersion surrounding a pRI limit are more likely “abnormal,” as they cannot be attributed to biological or analytical variation (with 95% probability).

To calculate the upper and lower EMU or dispersion limits surrounding a diagnostic result, pRI limit, or threshold, the following equations may be used:


$${\mathrm{EMU}}_{\text{Limit}}={\text{result}}_{\left(\text{concentration}\right)}\pm ({\text{result}}_{\left(\text{concentration}\right)}*{\mathrm{EMU}}_{\%})$$

or


$${\text{Dispersion}}_{\text{Limit}}={\text{result}}_{\left(\text{concentration}\right)}\pm ({\text{result}}_{\left(\text{concentration}\right)}*{\text{dispersion}}_{\%})$$

**Figure 1:**
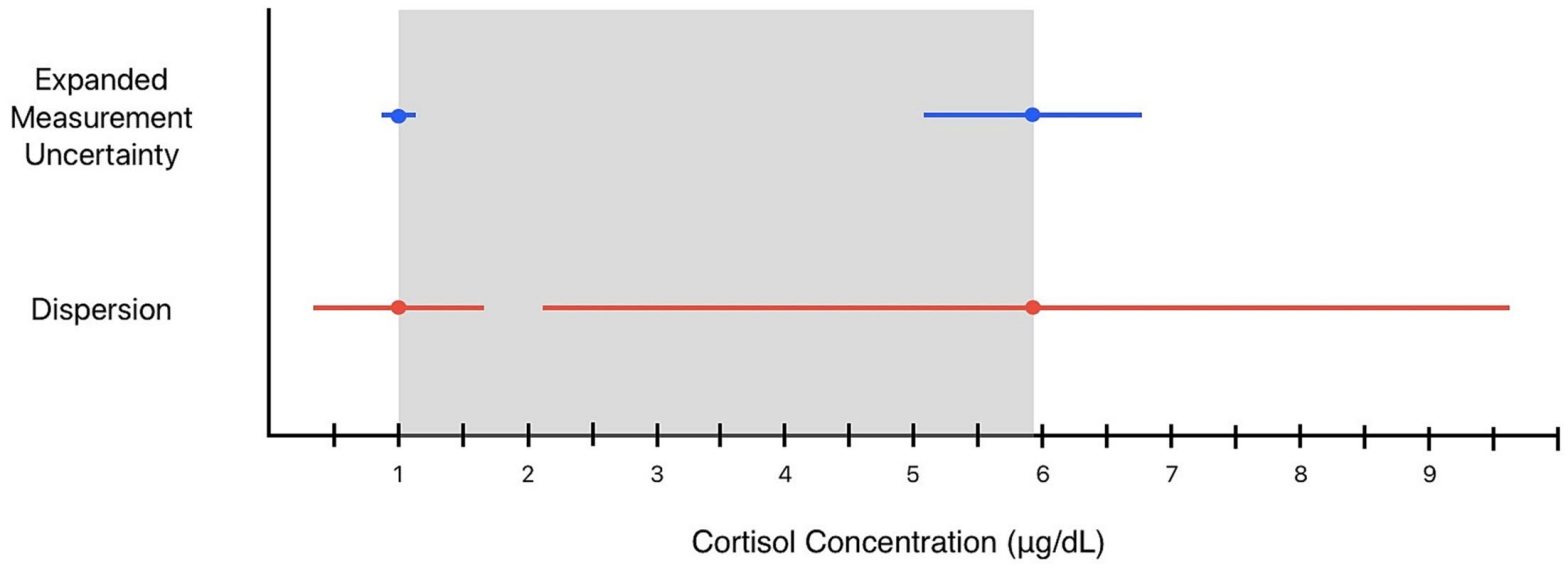
Expanded measurement uncertainty (blue lines) and dispersion (red lines) surrounding the population reference interval limits (grey box) of baseline cortisol in dogs with 95% probability. The expanded measurement uncertainty is 13.7%, resulting in a range of 0.86–1.14 μg/dL around the lower pRI limit of 1.0 μg/dL and a range of 5.1–6.7 μg/dL around the upper reference interval limit of 5.9 μg/dL. The dispersion is 64.9%, resulting in a range of 0.35–1.7 μg/dL around the lower pRI limit of 1.0 μg/dL and a range of 2.1–9.7 μg/dL around the upper reference interval limit of 5.9 μg/dL. Concentrations within these limits are of uncertain significance, as they could represent changes explained by analytical and/or biological variation with 95% probability.

Figure 1 illustrates EMU and dispersion around the upper and lower limits of our laboratory’s pRI for baseline cortisol (pRI: 1.0–5.9 μg/dL). When EMU (±13.7%) is applied to the lower reference limit of 1 μg/dL, it results in a margin of ±0.14 μg/dL. This “grey zone” spanning from 0.86–1.14 μg/dL represents a range of possible measurements that may be considered equivalent to 1 μg/dL with repeated measurements when accounting for analytical variability. A decreased cortisol concentration of 0.9 μg/dL could be attributable to analytical variability and may not represent a true concentration below the pRI. On the other hand, a measured cortisol value of 0.8 μg/dL would fall outside this range and cannot be attributed to analytical variation. The EMU surrounding the upper reference limit of 5.9 μg/dL results in a margin of ±0.81 μg/dL, or a “gray zone” spanning concentrations from 5.1–6.7 μg/dL.

When dispersion of 64.9% is applied to the pRI for cortisol, it provides a range of uncertainty surrounding the lower reference limit from 0.35—1.7 μg/dL. In other words, the clinical significance of a single cortisol concentration between 0.35—1.7 μg/dL is uncertain. The dispersion around the upper limit of our institution’s pRI encompasses a wide range of cortisol concentrations between 2.1—9.7 μg/dL. Concentrations within this range could reflect a “true” HSP either above or within the reference interval with repeated analysis. Two theoretical examples are provided to illustrate the clinical utility of dispersion.

**Figure 2:**
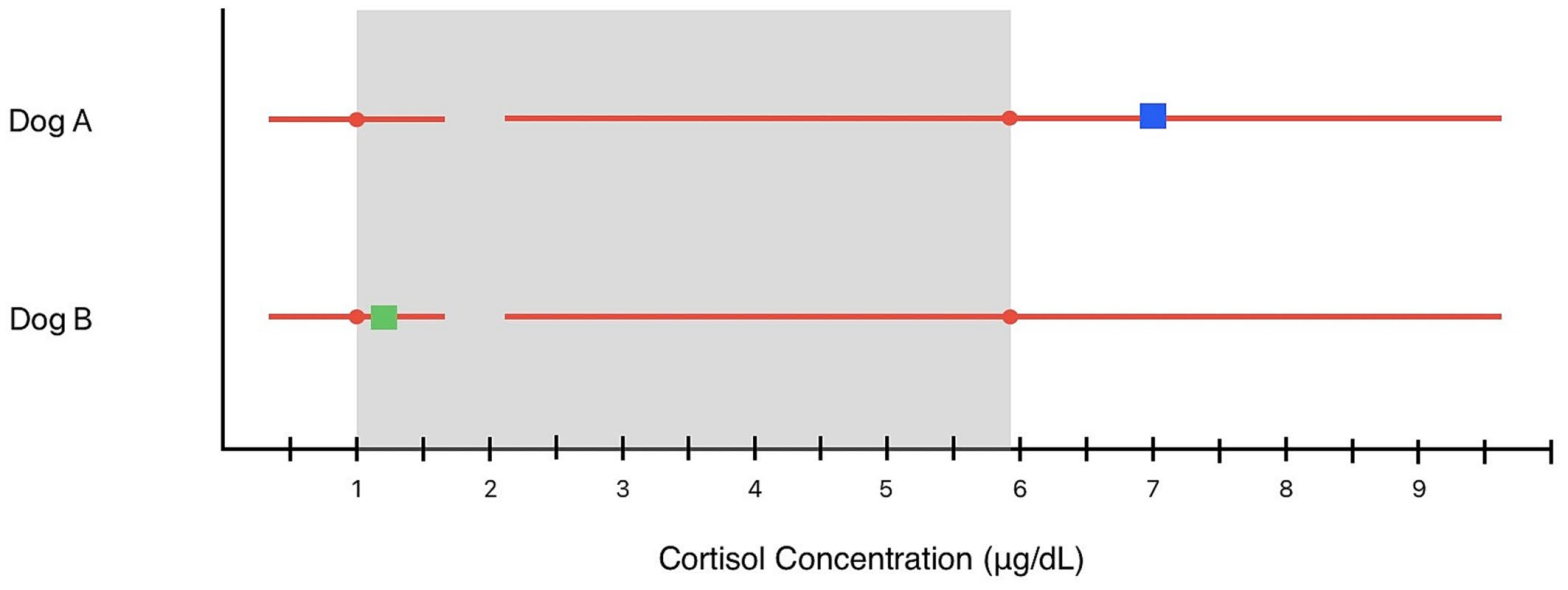
Examples of interpretation of patient results using dispersion around population reference interval limits. Dispersion is represented by the RED lines extending from the pRI limits. The gray box represents the pRI range for the institution. The dispersion is 64.9%, resulting in a range of 0.35–1.7 μg/dL around the lower pRI limit of 1.0 μg/dL and a range of 2.1–9.7 μg/dL around the upper reference interval limit of 5.9 μg/dL. The result for “Dog A,” 7.0 μg/dL (blue box), is above the pRI but within the dispersion and is of uncertain clinical significance. This may represent a “normal” homeostatic setpoint and should be interpreted within the clinical context. The result for “Dog B,” 1.2 μg/dL (green box), is within the pRI and the dispersion around the lower pRI. The clinical significance of this single result is uncertain, and further testing should be pursued if hypoadrenocorticism is suspected, even though the result is within the pRI.

Example of using dispersion to interpret baseline cortisol concentrations (Figure 2):

A clinically healthy dog has a baseline cortisol concentration of 7 μg/dL, which exceeds the institutional range of 1.0–5.9 μg/dL (27.6–162.84 nmol/L) (Figure 2, “Dog A”). What is the clinical significance of this finding?

At first glance, this result above the pRI might suggest some degree of cortisol dysregulation, leading the veterinarian to recommend further testing (e.g., a dexamethasone suppression test or ACTH stimulation test). However, this value falls within the dispersion around the upper pRI. While this baseline cortisol could reflect a pathological change to the HSP, it is also possible that this dog is physiologically normal and the mild elevation is due to biological and analytical variation. A veterinarian need not automatically recommend additional testing when a baseline cortisol concentration is outside of the pRI but within the dispersion. A single basal cortisol greater than 9.7 μg/dL would represent a HSP outside of the pRI with 95% probability.

A veterinarian has just measured baseline cortisol in a dog with suspicion of hypoadrenocorticism. The cortisol concentration of 1.2 μg/dL is within the pRI of 1.0–5.9 μg/dL (27.6–162.84 nmol/L) (Figure 2, “Dog B”). What is the clinical significance of this finding?

While this result is within the institutional pRI for cortisol, it is also within the dispersion around the lower pRI. The clinical significance of this result is uncertain, and this could possibly represent a “true” HSP for cortisol below the pRI. Additionally, this value is below the clinically significant cutoff of 2 μg/dL often suggested as a guide to determine if further investigation by ACTH Stimulation Test is needed (55 nmol/L) (1–4). Although this test result is within the pRI, additional investigation of hypoadrenocorticism should be pursued if clinically indicated.

Summary of EMU and dispersion for baseline cortisol concentrations:

EMU and dispersion are evidence-based, interpretive tools to account for analytical and biological variation in a measurand result. This approach is especially useful for measurands with significant biological fluctuation such as cortisol. The dispersion around baseline cortisol pRI limits is wide, and a single result within the dispersion is of uncertain significance. Veterinarians should not rely only on a pRI for cortisol, reinforcing current consensus statements recommending interpretation of baseline cortisol in the context of other clinical observations and ACTH stimulation or dexamethasone suppression test results (11). Incorporating variation—via EMU or DI—into interpretation frameworks helps shift clinical thinking away from dichotomous (normal/abnormal) conclusions and toward an individualized approach to laboratory result interpretation.

### Estimation of homeostatic setpoint and index of individuality

As the dispersion around a single cortisol measurement is wide, a veterinarian might wonder how many samples would be required for an accurate estimate of the homeostatic setpoint. The number of samples required to determine the HSP for cortisol with 95% confidence was calculated using the following formula (5, 9):


$$\#\text{samples}={\left(Z*\frac{\sqrt{{\mathrm{CV}}_{I}^{2}+{\mathrm{CV}}_{A}^{2}}}{\mathrm{Dev}}\right)}^{2}$$

Here, Dev represents the desired percentage deviation from the true HSP which was set at 10%. Generally, 10% allowable deviation is chosen for measurands with CV_I_ ≥ 6.67. For measurands with CV_I_ < 6.67, recommended Dev = 1.5 × CV_I_ (up to a maximum Dev of 10%, corresponding to CV_I_ = 6.67). Z is the standard deviation corresponding to the chosen confidence level (1.96 for 95% confidence) (9). Using CV_A_ = 6.86% and CV_I_ = 32.41% as found in Part 1, 42 samples are required to estimate the homeostatic setpoint of baseline cortisol in a patient with 95% confidence. This is impractical in most clinical situations as it requires multiple blood samples collected over an extended period each year. Individualized reference intervals formulated by estimating a patient’s homeostatic setpoint is a cumbersome option for monitoring a dog’s cortisol concentration over time.

The index of individuality gives a ratio of between-subject variation (group variation, or CV_G_) to within-subject (individual) and analytical variation (CV_I_ and CV_A_) to assess whether population-based reference intervals or personalized tools such as the reference change value (RCV) are more appropriate for patient data interpretation. The index of individuality (IoI) was calculated using the following formula (5, 8):


$$\text{Index of Individuality}={\mathrm{CV}}_{G}/\surd ({\mathrm{CV}}_{I}^{2}+{\mathrm{CV}}_{A}^{2})$$

The result was used to evaluate the applicability of conventional pRIs according to prior recommendations (5, 8) - an IoI less than 0.7 was considered to indicate that traditional population-based reference intervals (pRIs) are appropriate for patient data interpretation, while an IoI greater than 1.7 was considered to suggest that individualized patient reference intervals or reference change values are more likely to detect significant changes in serial results. An intermediate IoI (between 0.7 and 1.7) was considered to indicate that both pRIs and individual biological variation tools should be evaluated.

Biological variability in baseline cortisol results in an intermediate IoI of 1.14. The intermediate IoI suggests that a combination of pRIs and individualized reference intervals (iRIs) or RCV should be utilized for patient data interpretation depending on the clinical scenario. Several methods of iRI development have been proposed, including the use of biological variation tools such as RCV (13). An example of using RCV to establish individualized thresholds for interpretation of baseline cortisol concentrations is provided below.

The large number of samples required to estimate the HSP and the moderate IoI for baseline cortisol support current consensus statements recommending interpretation of baseline cortisol concentrations and potential endocrinopathies within the full clinical context (11). Veterinarians should not rely only on the pRI, but should instead incorporate patient history, concordant clinicopathological data, diagnostic thresholds, and other biological variation tools to guide the need for ancillary diagnostic testing.

### Reference change value

The growing accessibility of point-of-care hormone analysis has made in-house cortisol measurement more common in veterinary practice, and confident interpretation of serial results is required. Additionally, the intermediate IoI suggests that the interpretation of a cortisol concentration should include previous results when available. The reference change value (RCV) represents the percentage of change between two consecutive results that is considered significant when accounting for individual and analytical variation.

The unidirectional RCV with 95% confidence was calculated using the following formula (5, 8):


$$\text{Reference Change Value}\;(\mathrm{RCV})=Z*\sqrt{2}*\surd ({\mathrm{CV}}_{A}^{2}+{\mathrm{CV}}_{I}^{2})$$

The factor 1.65 was used to correspond to a unidirectional Z-score for 95% probability. The RCV calculated from cortisol biological variation data indicates that a serial change in baseline cortisol concentration must be at least 77.27% (unidirectional) to be significant with 95% probability. In other words, if a dog’s baseline cortisol concentration has changed by less than 77% across two sequential measurements, the variation could be due to biological and analytical variation. Sequential changes exceeding 77% likely reflect a change in the dog’s HSP for cortisol. Additional bidirectional and unidirectional RCVs for 99, 95, 90, and 80% probability are provided in Table 2.

**Table 2:** Reference change value (RCV) expressed in % for cortisol result interpretation at different significance levels, presented for both unidirectional and bidirectional analysis.

Probability	Unidirectional				Bidirectional			
	Z	RCV	95% CI (lower)	95% CI (upper)	Z	RCV	95% CI (lower)	95% CI (upper)
99%	2.33	109.12%	78.24%	138.03%	2.58	120.83%	86.64%	152.88%
95%	1.65	77.27%	55.41%	97.77%	1.96	91.79%	65.82%	116.14%
90%	1.28	59.95%	42.98%	75.85%	1.65	77.27%	55.41%	97.77%
80%	0.84	39.34%	28.21%	49.77%	1.28	59.95%	42.98%	75.85%

Lower and upper 95% confidence intervals for the corresponding RCV are provided.

RCV may be used to establish a limit in units of the test using the formula:


$${\mathrm{RCV}}_{\text{Limit}}={\text{result}}_{\left(\text{concentration}\right)}\pm ({\text{result}}_{\left(\text{concentration}\right)}*{\mathrm{RCV}}_{\%})$$

Reference change factors may also be calculated for two or more serial results or for variation with non-normal distribution using log-normal equations as described by Lund, et al. (14).

Three theoretical examples of the clinical application of RCV to baseline cortisol are provided.

**Figure 3:**
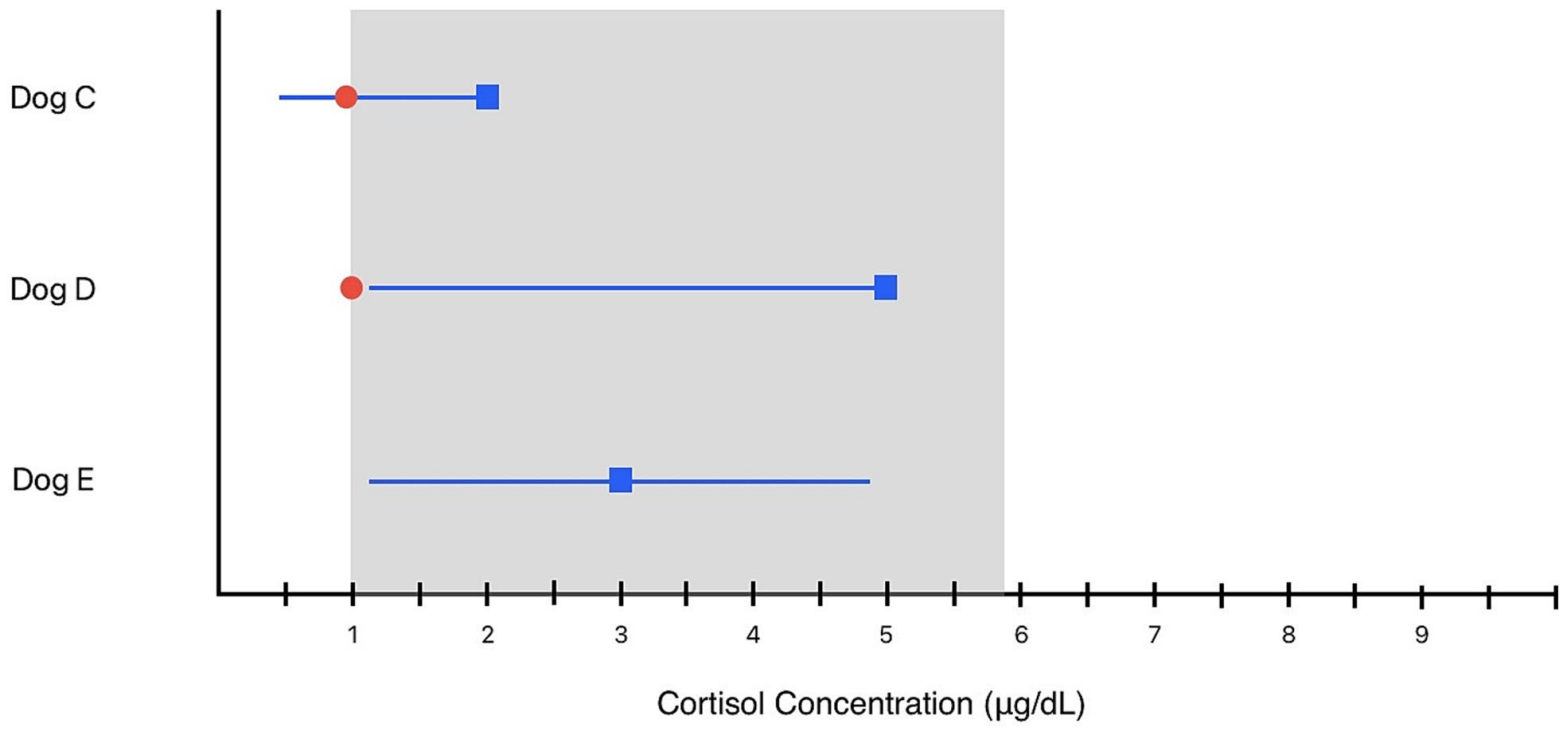
Examples of the interpretation of baseline cortisol concentration in dogs using reference change values. Blue boxes and the lines extending from the boxes indicate the prior test result and the unidirectional or bidirectional RV, the gray shaded area represents the institution’s population reference interval, and the red circle represents the most recent laboratory result. For “Dog C,” the unidirectional RCV with 95% probability from the previous result of 2 μg/dL has a lower limit of 0.46 μg/dL. The most recent result of 0.9 μg/dL is within the RCV and is of uncertain significance, although just outside the pRI. For “Dog D,” the unidirectional RCV with 95% probability from the previous result of 5 μg/dL has a lower limit of 1.15 μg/dL. The most recent result of 1 μg/dL represents a significant change that cannot be attributed to biological and analytical variation with 95% probability, and further investigation is warranted. For “Dog E,” bidirectional RCV with 80% probability is used to establish limits around the previous measurement of 3 μg/dL.

Examples of using RCV to interpret baseline cortisol concentrations (Figure 3):

A veterinarian has decided to include baseline cortisol concentrations with their wellness biochemistry panels. A clinically healthy dog has a recent baseline cortisol of 0.9 μg/dL, outside the laboratory pRI of 1.0–5.9 μg/dL (Figure 3, “Dog C”). The prior baseline cortisol in this individual was 2 μg/dL. Does this represent a change in the HSP with 95% confidence?

When the unidirectional RCV of 77% is applied to the previous concentration of 2 μg/dL, the RCV threshold is 0.46 μg/dL. In other words, a baseline cortisol below 0.46 μg/dL would be required to be confident of a change in the HSP with 95% confidence. Changes between 0.46–2 μg/dL are of uncertain significance and could be attributed to biological and analytical variation. Continued monitoring without immediate ancillary testing or further diagnostic investigation are both reasonable clinical actions depending on the degree of suspicion for illness, financial constraints, owner and veterinarian comfort with uncertainty, and other factors.

A different dog with signs suggesting hypoadrenocorticism within the same practice has a baseline cortisol of 1 μg/dL. The prior concentration obtained during health was 5 μg/dL (Figure 3, “Dog D”). Both serial measurements are within the pRI. Is this change significant with 95% probability? When a unidirectional RCV is applied to the previous measurement of 5 μg/dL, we find a lower RCV limit of 1.15 μg/dL. The change in baseline cortisol cannot be attributed to biological and analytical variation. While the result is at the pRI limit, it likely represents a true change in the HSP for this patient and warrants further investigation, especially considering the clinical suspicion of hypoadrenocorticism.

While both dogs have similar baseline cortisol concentrations near the lower pRI limit with a single measurement, the clinical interpretation differs based on serial testing and patient history. This example illustrates how RCV can be incorporated as a component of clinical decision making.

A bidirectional RCV may be used to establish individualized limits around a baseline cortisol measurement. If the next cortisol concentration is beyond these limits, it suggests a significant change that might warrant further investigation. A veterinarian might lower the desired probability in order to alert them to changes that may warrant further investigation.

For example, a baseline cortisol in a dog during wellness testing is 3 μg/dL (Figure 3, “Dog E”). The veterinarian would like to set RCV thresholds that might indicate a significant change in the dogs’ HSP with 80% probability for use alongside the pRI to interpret a future cortisol measurement. What are the bidirectional RCVs with 80% confidence around the cortisol concentration of 3 μg/dL?

The bidirectional RCV with 80% confidence for baseline cortisol is 60.0% (Table 2). When applied to the test result of 3 μg/dL, we calculate a lower RCV threshold of 1.2 μg/dL and an upper RCV threshold of 4.8 μg/dL. In other words, a subsequent baseline cortisol between 1.2–4.8 μg/dL could be reasonably attributed to biological and analytical variation. A result beyond these RCVs for this patient suggests a change in the HSP with 80% probability and may be worth additional investigation.

## Discussion

Appropriate interpretation of most clinical tools and tests is nuanced. Biological variation tools are no exception, and several important limitations merit brief discussion. First, the calculated values in this manuscript are specific for baseline cortisol concentrations in dogs and should not be applied to cortisol concentrations obtained after administration of ACTH, exogenous glucocorticoids, medications that affect cortisol homeostasis such as trilostane, or in dogs with previously diagnosed cortisol dysregulation. Second, it is important to emphasize that BV tools describe the expected repeatability of laboratory measurements, but do not provide direct estimates of disease probability for a given data point. Finally, BV tools should be cautiously applied when estimating the probability of disease using diagnostic cut-offs or decision limits where the test performance has been previously described.

Cortisol concentrations exhibit significant circadian rhythms and pulsatile secretion patterns in dogs and people through the intricate homeostatic mechanisms of the hypothalamic–pituitary–adrenal (HPA) axis (15, 27, 28). While many biochemical measurands have been shown to have similar CV_I_ in health and stable disease (16), this may not be true for baseline cortisol. In people, hyperadrenocorticism has been shown to alter this rhythmic control and decrease the coefficient of variation of the amplitude of cortisol secretory episodes (17, 29). Administration of medications affecting the HPA axis also may affect variability in cortisol secretory patterns. In dogs, less significant biological variability (CV_I_ and CV_G_) has been reported for cortisol concentrations after administration of ACTH (18). The impact of cortisol homeostatic dysregulation due to disease or medication administration on biological variation in dogs is unknown, and the application of biological variation tools derived from CV_I_ and CV_G_ in healthy dogs may not be appropriate in these situations. Data regarding the biological variation of baseline cortisol concentrations in healthy dogs should not be applied to post-ACTH or post-dexamethasone dynamic testing results, in dogs receiving exogenous glucocorticoids or medications that directly affect steroidogenesis (e.g., trilostane), or in patients with previously diagnosed hyperadrenocorticism or hypoadrenocorticism.

**Figure 4:**
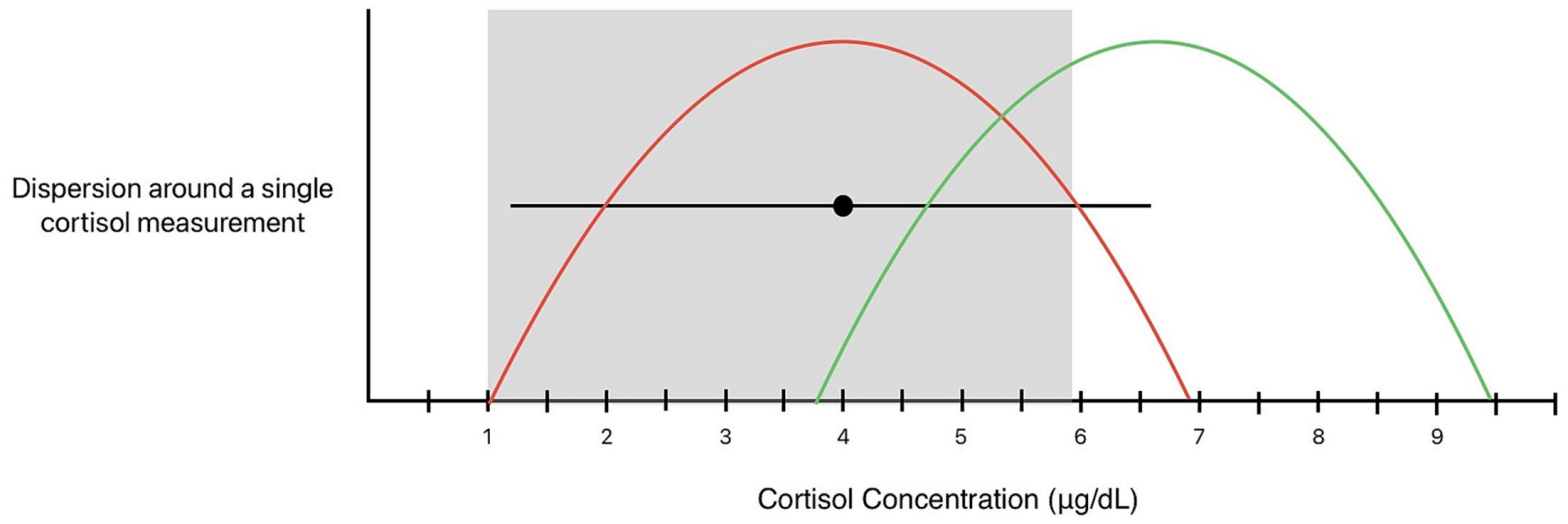
A bidirectional dispersion around a single cortisol measurement of 4 μg/dL with 95% probability. The black dot represents a single numerical measured value, the black lines illustrate the dispersion around this result, and the grey box illustrates the population reference interval. The red curve illustrates a theoretical distribution of repeated measurements, and future measurements are expected to fall within the dispersion with 95% probability (95% of future samples). When considering dispersion in terms of the “true mean” (or HSP), 4 μg/dL could represent the mean (red curve) or could represent a single measurement away from the mean (green curve).

While biological variation tools provide an evidence-based answer to the question, “how repeatable is the result?” they do not directly answer the question, “what is the likelihood this value represents the ‘true’ mean/HSP?”. They consider that the measured value could represent the “true” mean (or HSP) or some measurement away from the “true” mean, even if the latter scenario is less likely (Figure 4). Indeed, if repeated measurements are normally distributed around the mean or HSP, the single, most likely “true” result is the measurand value itself. While dispersion provides a range in which the “true” HSP may lie, and RCV provides a range within which a change in a serial measurement could be explained by biological and analytical variation, these tools do not directly predict the likelihood that the true HSP lies outside a chosen threshold (pRI) or the likelihood of a significant HSP change when associated with single measurand values within the given range.

**Figure 5:**
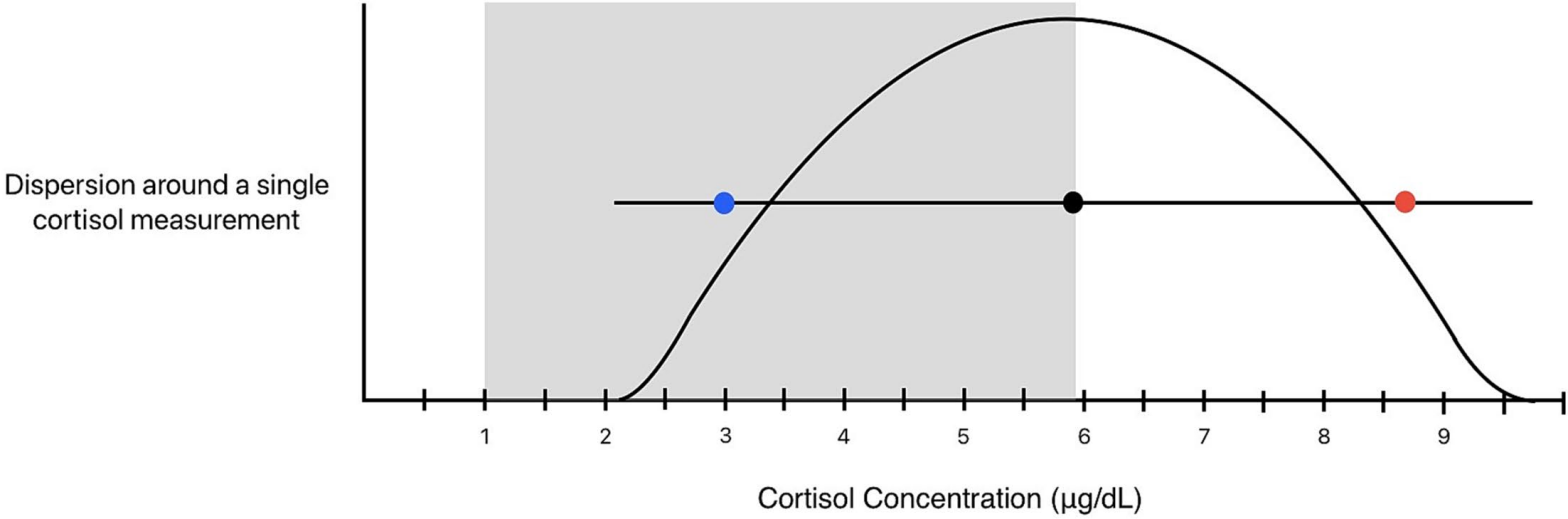
The dispersion for baseline cortisol around the upper population reference interval limit of 5.9 μg/dL (black dot) spans values from 2.1–9.7 μg/dL. The black curve illustrates a theoretical distribution of repeated measurements around the upper pRI. Baseline cortisol of 3 μg/dL (blue dot) and 8.8 μg/dL (red dot) both lie within the dispersion, but these values do not carry the same inferential weight. A concentration of 8.8 μg/dL is more likely to represent a true elevation beyond the pRI than a concentration of 3 μg/dL.

This distinction can be illustrated when interpreting a result using the dispersion for baseline cortisol concentrations around the upper limit of the pRI (Figure 5). The dispersion around our institution’s upper pRI of 5.9 μg/dL encompasses values ranging from 2.1—9.7 μg/dL. A single baseline cortisol concentration of 3 μg/dL or 8.8 μg/dL are both within the dispersion of the upper reference interval. In other words, both values are expected measurements (with 95% probability) that could be obtained if a sample with a true mean (or HSP) of 5.9 μg/dL were repeatedly analyzed. However, a cortisol concentration of 8.8 μg/dL is more likely to represent a true HSP outside the pRI than a cortisol concentration of 3 μg/dL. While they are both within the dispersion, these values have different likelihoods of representing an “abnormal” HSP.

While the distinction between measurement repeatability and predictive performance may seem pedantic, it becomes important when biological variation tools are applied to validated decision limits. The predictive value of a validated decision limit is found by combining the prior probability of disease for an individual patient with the sensitivity and specificity of the test as it performed against a gold standard. For continuous variables, a receiver operating characteristic (ROC) curve is commonly used to identify cut-offs for a binary classification model. As measured values become more extreme, their ability to predict disease or outcome generally improves. Variability inherent to the study population and analytical method is embedded within the observed diagnostic performance and contributes to the shape and accuracy of the ROC curve. Although variance within the dataset is infrequently reported, it is implicit within the data and influences overall test performance.

Several retrospective studies have assessed the sensitivity and specificity of single baseline cortisol concentrations for the diagnosis of hypoadrenocorticism in dogs using the ACTH stimulation test as a gold-standard for diagnosis (1–4). The sensitivity and specificity reported for a single baseline cortisol concentration above 2 μg/dL ranged from 93 to 100% and 63–78%, respectively. When diagnostic cut-offs and decision limits are applied to patient data, veterinarians should incorporate the test performance (sensitivity and specificity) with the prior probability of disease to estimate the post-test probability of disease (predictive value) (19–21). While the variance of a measurand can also be incorporated into a probabilistic framework to further refine the predictive value, such models and their validation are beyond the scope of the present manuscript. Assumptions regarding the variance of the reference population would be necessary for such models.

While the degree to which biological variation affects prior decision thresholds is not always apparent, the potential impact of new analytical methods on previously established decision limits cannot be ignored. Korchia and Freeman (22) provide a clear and comprehensive summary of the chronological and technical evolution of assays used to measure serum cortisol in dogs. They highlight that the commonly used cortisol cut-offs of 1.4 μg/dL and 20 μg/dL (38.6 nmol/L and 552 nmol/L, respectively) for diagnosis of HAC with a dexamethasone suppression test were originally established in 1983 using a competitive protein-binding method, an assay that is no longer in routine use (23, 24). Although interpretation thresholds are still widely used for both the ACTH stimulation test and the low-dose dexamethasone suppression test (LDDST), these thresholds have not been fully re-evaluated and their diagnostic accuracy (sensitivity and specificity optimized via ROC curve analysis) has not been definitively characterized in the context of more modern platforms such as the Immulite 2000 Xpi.

As cortisol assay methodologies have evolved significantly over the past decades, it is essential to consider analytical differences when transferring historical cut-offs to newer systems. This is of particular concern in borderline cases where small shifts in measured values can affect diagnostic interpretation. Recent veterinary and clinical laboratory studies have highlighted the importance of establishing method- and instrument-specific RIs whenever possible, in accordance with guidelines such as those from the ASVCP and the CLSI. If in-house validation is not feasible, laboratories should assess analytical bias by comparing their results to those obtained with a validated reference method or platform. If a significant bias is present, results should be harmonized or decision limits adjusted to avoid diagnostic errors (13, 25, 26).

The impact of biological variation on diagnostic cut-offs is also important when considering expert or consensus opinions without reported diagnostic accuracy, such as those used to categorize disease severity. While these opinions are helpful in clinical decision-making, the uncertainty and predictive potential associated with a proposed cut-off is usually poorly defined. While it is not possible to objectively quantify uncertainty around these limits, considering the EMU or dispersion around a proposed cut-off may provide a clinically relevant starting point. Consensus-derived decision limits may benefit from establishing a “grey-zone” around cut-offs utilizing analytical or biological variation data to provide veterinarians with the tools needed to contribute to individualized spectrum-of-care.

## Conclusion

Biological variation tools for baseline cortisol concentrations in dogs have been calculated and presented. These tools are intended as interpretive aids to assist in evaluation of unexpected results or results close to reference interval limit and avoid misclassification of cases. Tools such as EMU, dispersion, and RCV encourage a trichotomous framework for interpreting laboratory results: normal, abnormal, or indeterminate. This aligns with broader trends in laboratory medicine that aim to reduce misclassification, enhance clinical decision-making, and personalize diagnostic interpretation. The calculated IoI and HSP for baseline cortisol reinforce prior expert consensus statements encouraging interpretation of baseline cortisol in conjunction with clinical signs and dynamic testing such as the dexamethasone suppression test or the ACTH stimulation test rather than reliance on population reference intervals alone. Biological variation tools assist veterinarians when practicing individualized spectrum-of-care, especially with regards to complex, variable biomarkers like cortisol.

## Data Availability

The original contributions presented in the study are included in the article/supplementary material, further inquiries can be directed to the corresponding author.
